# Toward a Responsibility-Catering Prioritarian Ethical Theory of Risk

**DOI:** 10.1007/s11948-018-0036-2

**Published:** 2018-03-05

**Authors:** Per Wikman-Svahn, Lars Lindblom

**Affiliations:** 1 Department of Philosophy and History, KTH Royal Institute of Technology, Teknikringen 76, 100 44 Stockholm, Sweden; 2 Department of Historical, Religious and Philosophical Studies, Umeå University, Humanisthuset, 100 44 Umeå, Sweden

**Keywords:** Philosophy of risk, Ethics, Responsibility, Responsibility-catering prioritarianism, Risk regulations, Risk management

## Abstract

Standard tools used in societal risk management such as probabilistic risk analysis or cost–benefit analysis typically define risks in terms of only probabilities and consequences and assume a utilitarian approach to ethics that aims to maximize expected utility. The philosopher Carl F. Cranor has argued against this view by devising a list of plausible aspects of the acceptability of risks that points towards a non-consequentialist ethical theory of societal risk management. This paper revisits Cranor’s list to argue that the alternative ethical theory responsibility-catering prioritarianism can accommodate the aspects identified by Cranor and that the elements in the list can be used to inform the details of how to view risks within this theory. An approach towards operationalizing the theory is proposed based on a prioritarian social welfare function that operates on responsibility-adjusted utilities. A responsibility-catering prioritarian ethical approach towards managing risks is a promising alternative to standard tools such as cost–benefit analysis.

## Introduction

The characterization of risks raises ethical issues. Risks are often defined based on only probabilities and (unwanted) consequences (Aven 2012). This definition is, for example, prevailing in the standard methods of probabilistic risk analysis (PRA). PRA is commonly used, and sometimes even part of the regulatory framework, in a wide range of sectors in society, including transportation, energy, construction, chemical industries, aerospace, the military, finance, accounting and business administration (Bedford and Cooke 2001).

But while this technical definition might be standard in quantitative treatments of risks, many have pointed out that the conceptualization of risk ought to be much more complicated. Social scientists have gathered abundant empirical evidence that people’s perception of risks depends on a multitude of factors (e.g., Lupton 1999; Zinn 2008; Slovic et al. 2009). Philosophers have made convincing arguments that risks are not objective entities that exist on their own, but exist in a context that ought to influence how one acts concerning risks (e.g., Hermansson and Hansson 2007; Hansson 2013).

The view of risks as probabilities and consequences is also typically used in related quantitative tools for economic analysis of risks such as cost–benefit analysis (CBA) (or risk–benefit analysis). CBA is increasingly used in used in regulatory settings such as workplace health and safety, traffic regulations, pollution law and even anti-trust policy (Adler and Posner 2006; Sunstein 2002). CBA can be seen as a practical application of the ethical theory of consequentialism—the idea that the overall consequences of an action determine what is right or wrong (Hansson 2007). The paradigmatic form of consequentialism is utilitarianism—the ethical view that an act is morally right if and only if that act maximizes the total utility (Sinnott-Armstrong 2015).

But the idea that social policies towards risks can be justified by a utilitarian ethics based on a calculation of probabilities and consequences has also been much criticized. For example, philosophers have noted that cost–benefit analysis has difficulties accommodating ethical intuitions, such as when and why risks are acceptable and how distributive concerns should be taken into account (e.g., Hansson 2007; Wolff 2006).

The philosopher Carl F. Cranor is a long-time critic of the prevailing views of risks in public risk management policies (e.g., Cranor 1995, 1997, 2007, 2009). Cranor’s (2007) article *Toward a non*-*consequentialist approach to acceptable risks* lists nine aspects of acceptability of risks that “seem sufficiently plausible that any defensible ethical view should be able to provide reasons to justify them” (p. 37). Cranor suggests that these aspects should be part of “a more plausible approach to how we should think about risks and their acceptability” (p. 41). Cranor is critical of the justification of social policies towards risks on utilitarian ethical grounds and argues that there are non-consequentialist ethical theories (especially contractualism and deontology) that are better suited for handling the kinds of aspects in his list. Cranor acknowledges that sophisticated utilitarian accounts might be more able to accommodate the elements of his list.

This paper revisits Cranor’s article but proposes an alternative to both standard utilitarian and Cranor’s non-consequentialist approaches. The proposed approach is based on an ethical theory developed in political philosophy called *responsibility*-*catering prioritarianism*, which combines the values of efficiency, responsibility, and concern for those worst off, and instructs us to maximize moral value understood as a function of those values.

It will be argued that responsibility-catering prioritarianism can accommodate the challenges identified by Cranor, and at the same time handle other requirements of an ethical theory of risk. Moreover, it is proposed that responsibility-catering prioritarianism can be operationalized in a social choice framework with a prioritarian social welfare function that gives more weight to the worse off. This social welfare function operates not on standard individual utilities, but on what is called responsibility-adjusted utilities. Responsibility-adjusted utilities reflect the utility that could normally be expected for individuals, taking into account circumstances outside the control of the individual.

The paper is structured as follows: in “Plausible Requirements of an Ethical Theory of Risk”, Cranor’s list is examined along with other aspects from the literature to identify the basic ethical requirements of an ethical theory of risk. “Introducing Responsibility-Catering Prioritarianism” introduces the theory. “Responsibility-Catering Prioritarianism and the Basic Requirements of an Ethical Theory of Risk” returns to Cranor’s list and shows how it can be used to inform a responsibility-catering prioritarian theory of risk. “Operationalizing the Theory” turns to the more practical issue of how to implement responsibility-catering prioritarianism in practice and proposes that a social choice framework can be used to operationalize the theory. The article ends with a “Concluding Discussion”.

## Plausible Requirements of an Ethical Theory of Risk

Cranor (2007) lists nine aspects of acceptability of risks that builds on previous accounts of the acceptability of risk (Cranor 1995). Cranor argues that these aspects significantly differ from the prevailing view of risks and risk management in the technical community. The list of aspects identified by Cranor provides insights into the fundamental requirements of an ethical theory of risk, but the elements in the list can be characterized more simply. Moreover, there are additional aspects found in other literature on the ethics of risk that are not emphasized by Cranor’s list.^1^

The first point in Cranor’s list is (1) the need to distinguish between risks that are imposed on a person and risks that the person takes. Cranor notes that these ideas often are conflated in discussions on risks by assuming that every risk is “taken”. This distinction between risks an agent chooses and risks that are imposed on the agent is necessary since a higher level of risk seems more acceptable for risks that are taken than for risks that are imposed.

Cranor’s second point is that one should distinguish between (2) risks that it is permissible for individuals to take in their own lives and risks that people are required to live with because they are a result of public policy. He gives the example that risks from recreational activities (such as mountain climbing or scuba diving) should not be used as a justification for other sources of risks to the general public, which are not, in this sense, chosen.

The third point is (3) degrees of control over risks. Protection against risks that are under an individual's control can, with moral justification, be left to a higher degree to the individual than risks that are not under an individual’s control. Cranor also points out that people often intuitively see a fundamental difference between (4) risks from natural phenomena and risks from human activities. When it comes to (5) the transparency of risks, one should be able to differentiate between risks that are identifiable, and thereby exercise self-protection and control over them, and risks that cannot be easily identified. The sixth point is the distinction between (6) voluntarily incurred and non-voluntarily risks. All things being equal, the former is more acceptable than the latter. To take a risk voluntarily “one must properly understand and be aware of the risks one is incurring, be competent to make decisions about the risks, and in some robust sense have consented or agreed (explicitly or implicitly) to them” (Cranor 2007, p. 40).

Cranor is right that these six points are reasonable aspects of the acceptability of risk, but there is something common in all six points: people should, to some degree, be held responsible for the risky choices they make, but not for risks that are not the results of their choices. For example, the reason that there is a difference between risks that are imposed and those that are taken is that in the latter case there is an element of choice for the person affected by the risk, which is absent in the former case. Also, risks created by human activities tend to be more controllable or avoidable than natural hazards, and natural risks can also be either reduced or amplified by human decisions. A high level of risk that is caused by choices one makes in daily life is more acceptable than an equally high level of risk that one is required to live with under public policy. Apparently, if one has control over a risk, one can exercise choice in handling the risk. However, one cannot know how to choose if one is ignorant of the options, consequences, or even the necessity of taking action. This insight can be summed up in the slogan; rational, informed, and free choice incurs responsibility. The reverse is, of course, that people should not be held responsible for risks that are not freely chosen.

The final three points in Cranor’s list are; (7) the relationship between the person creating or imposing a risk and the person exposed to the risk. Cranor says that “[f]rom a moral point of view when one party creates a risk, and another bears the costs of it, this raises issues about the distribution of benefits and risks of an activity, as well as issues of externalities” (Cranor 2007, p. 39). Point number eight is that (8) the magnitude of harms, benefits and the probability of the materialization of each are relevant for the acceptability of risk exposure. For example, a large risk with minor benefits should be judged differently than an equally large risk with greater benefits. The final aspect has to do with (9) the relationship of the risks and benefits to one’s project or life plan. Cranor argues that an ethical theory of risk must be able to distinguish between risks that have to do to with things central to one’s life plans or even identity, and risks that are related to lesser interests. For instance, if being a mountaineer is a person’s core identity in life, then the risks of climbing high steep mountains are probably more acceptable to that individual than the risks of driving a car to the supermarket.

These three final aspects in Cranor’s list have relevance for what should be handled in a consequentialist evaluation of risks: the magnitude of harms and benefits and the probability of each materializing, the distributions of risks and benefits. It should also represent personal preferences, including those that are related to individual projects and life identity.

Cranor’s list points to relevant aspects of what is reasonable to demand of a comprehensive view on risks, but, it does not, however, provide a complete picture. The list focuses on the weakest parts in the standard form of consequentialism, but aspects related to efficiency are missing from Cranor’s list. For example, it should matter if ten persons, 1000 persons, or 10 million persons are each exposed to a small risk, even if the risk to the most exposed individuals is the same (see, e.g., Adler 2005). One essential requirement of efficiency is the principle that if at least one individual is better off in one outcome than in another outcome and everybody else is on the same level, then the first outcome should be considered better than the second outcome. This requirement of efficiency can be formalized by principles such as the “Pareto-principle” or the “principle of personal good” (see e.g., Broome 1991).

Another aspect that is important, but is not emphasized in the list is the ethical requirements related to equity. An equal distribution of risks to different individuals is preferable to an unequal distribution (Keeney 1980; Fishburn 1984). For example, if two policies expect the same number of cancer cases, but in the first, the risks are spread more evenly than in the second, then the element of equity should count as a reason for choosing the first policy.

Thus, both efficiency and equity are significant requirements of an ethical theory of risk. However, what happens if policies differ regarding both efficiency and equity? Lenman (2008) illustrates this problem:

Assume that the current situation (the status quo) is that in a population of 20 million people, each faces a 1 in 500,000 risk of death. For some given cost, we may do one of two things:

Policy A: Reduce the risk to each of the 20 million people to 1 in 1 million.

Policy B: Reduce the risk to each of 19 million people to 1 in 19 million while the risk to the remaining million is increased to 1 in 100,000.

The expected number of fatalities for the status quo is 40. Policy A reduces this number to 20, while Policy B reduces it to 11. In this example, one policy (Policy B) is more efficient in reducing the expected number of fatalities, whereas the alternative (Policy A) renders a more equitable result. How should one choose? Many real policy options differ regarding both efficiency and equity, which makes the problem highly relevant. It is, therefore, reasonable to also demand that an ethical theory of risk should be able to evaluate policy options in a way that can take into account both the requirements of efficiency and equity.

In summary, it has been argued that the points in Cranor’s list provide clues to the ethical requirements of acceptability of risk. The first six points boiled down to issues of choice and responsibility, while the final three points have to do with what should be counted in a consequentialist evaluation. This is an insight that will be returned to in “Responsibility-Catering Prioritarianism and the Basic Requirements of an Ethical Theory of Risk”. It has also been argued that efficiency and equity are further relevant ethical requirements that need to be recognized. The next section presents an ethical theory that can incorporate all the aspects in Cranor’s list plus the additional requirements of efficiency and equity.

## Introducing Responsibility-Catering Prioritarianism

Responsibility-catering prioritarianism is well positioned to deal with the ethical requirements of efficiency because it advocates actions that maximize moral value. It also incorporates the demands related to equity by assigning a higher moral value to persons with lower well-being, while allowing for trade-offs between efficiency and equity. Moreover, it does so while taking responsibility into account. The theory was developed within the debate on distributive justice over the last decades by combining two previous philosophical discourses: The first is best characterized by Ronald Dworkin’s seminal writings on the equality of resources (1981, 2000). The second is epitomized by Derek Parfit’s (1997) introduction of the ethical theory of prioritarianism, which can be seen as a middle road between strict egalitarianism and strict utilitarianism. The synthesis of the two strands has in particular been promoted by Richard Arneson who also coined the term responsibility-catering prioritarianism. Both developments will be discussed in this section.

The contemporary debate in political philosophy started with the publication of John Rawls’s *A Theory of Justice* (1971), in which he argued that distributive egalitarian justice should be concerned with the basic structure of society (the framework of institutions in a society). While arguing for this approach, he also claimed that responsibility should not play a part in the conceptualization of equality. In opposition to John Rawls, Dworkin (1981, 2000) argued that choice should play a role in the conceptualization of equality. This was the start of an on-going debate on responsibility in political philosophy. Why did Dworkin think that responsibility and equality must go together? One reason is that one would not want to compensate people for their frustrated preferences for inequality to achieve equality in preference satisfaction.

More important, for both Dworkin’s purposes and the aims of this article, is the difference between inequalities that result from luck and those that result from choice. On the one hand, if I choose to give you an apple, or if you work for that apple as wages, it would be unfair to demand that I get it back for reasons of equality. On the other hand, if I happened to find a good apple tree by chance, it would be unfair of me not to share these apples with you. To clarify these different causes for inequality, Dworkin makes a distinction between brute luck and option luck:

Option luck is a matter of how deliberate and calculated gambles turn out—whether someone gains or loses through accepting an isolated risk he or she should have anticipated and might have declined. Brute luck is a matter of how risks fall out that are not in that sense deliberate gambles. (Dworkin 2000, p. 73)

The idea of option luck, then, may be characterized by a gambling analogy. If you choose to accept a risk, then it is fair that you bear the costs if the worst option materializes, just as it would be fair that you gained the profits if things turn out well. When it comes to brute luck, the element of choice is missing, and the concept of responsibility has no application. For Dworkin, equality then means that resources should be equitably distributed when they are the result of brute luck, but that differences in the allocation of resources resulting from option luck are fair. This distinction was a breakthrough in the debate on equality, and Dworkin’s seminal article (1981) was soon followed by others (Arneson 1989; Cohen 1989). For this paper, the critical point to note is that equity and responsibility can be combined, which also means that this kind of theory can incorporate two of the basic requirements of an ethical theory of risk as discussed above.

The second main development came with Parfit’s (1997) introduction of a middle way between egalitarianism and utilitarianism. Parfit pointed out that two intuitions seem at work when people try to justify equality. The first of these is that it is bad that some are worse off than others are. On this view, what is wrong with inequality is that the distribution of what is valuable is not the same to each person. The second intuition says that it is bad that some are worse off. From this perspective, what is wrong with an unequal distribution is that some people find themselves in adverse circumstances. Parfit argues that the first intuition is problematic because it gives rise to what he calls *the leveling down problem*.^2^ Parfit argues that the second intuition, or more specifically what he calls the *priority view*, should be investigated: that benefiting people matters more the worse off these people are.

Prioritarianism means that it is more important to benefit the worse off than the already well off. Notice however that this characterization does not say how much more important it is to benefit those who are worse off. For an infinite weight, then the result is a Rawlsian maximin. A zero weight results in utilitarianism (see also “Operationalizing the Theory”). In summary, this view solves the leveling down problem by taking efficiency into account, but it retains a fundamental principle commonly used to guide us towards equality. This kind of theory, therefore, incorporates a well-balanced tradeoff between equity and efficiency.

Insights gained from these developments were then used to create theories that combine notions of responsibility, efficiency, and equity in the “priority” sense. In particular, Richard Arneson has developed what he calls “responsibility-catering prioritarianism”, which combines Dworkin’s emphasis that choice matters with Parfit’s priority approach. Arneson characterizes responsibility-catering prioritarianism as follows:

Institutions and practices should be set and actions chosen to maximize moral value, with the moral value of achieving a gain (avoiding a loss) for a person being (i) greater the greater amount of well-being for the person gain (averted loss) involves, (ii) greater, the lower the person’s lifetime expectation of well-being prior to receipt of benefit (avoidance of the loss), (iii) greater, the larger the degree to which the person deserves this gain (loss avoidance). We ought to maximize well-being weighted by priority and responsibility. (Arneson 1999, pp. 239–240)

On this theory, then, a course of action is morally justified if it maximizes moral value, where such value is understood as being a function of responsibility, priority, and welfare. Responsibility-catering prioritarianism conceives of responsibility in terms of the distinction between brute and option luck. It defines the worst-off as those with the lowest level of welfare and demands that their interests should receive priority. Moreover, it takes account of responsibility and priority, while sharing the view with utilitarianism that the pursuit of human welfare is of fundamental moral importance. In the next section, it will be showed how responsibility-catering prioritarianism could deal with the basic requirements of acceptability of risk and how this theory, *pace* Cranor, can do so while remaining consequentialist.

## Responsibility-Catering Prioritarianism and the Basic Requirements of an Ethical Theory of Risk

It is easy to see that responsibility-catering prioritarianism incorporates the requirement of efficiency as it aims to maximize moral value. In some, but only some, situations responsibility-catering prioritarianism would likely give the same prescriptions as utilitarianism. For example, in situations where those who are affected by risks are equally well off and equally responsible for the situation. Moral value is then maximized by utility exclusively.

For equity, responsibility-catering prioritarianism entails that the moral value of outcomes varies with the distribution of utility between different individuals. This sensitivity to equity means that the theory is not vulnerable to one of the primary criticisms of utilitarianism (and standard versions of cost–benefit analysis. To see how this works, assume, for the sake of exposition, that money is a measure of welfare. In a case where either a rich person stands to lose $200 or a poor person stands to lose $150, and only one of them can be protected, standard utilitarianism would protect the assets of the rich person. However, prioritarianism implies that the welfare of the poor should be granted more weight. For instance, if it is at least twice as important to help a person avoiding becoming destitute, than protecting a loss to a person who will remain rich, then prioritarianism can provide the appropriate prescription. Granted, the question of what weight to give to equity is difficult, but there are approaches to find a solution to this problem. One can test both intuitions about risk and attitudes to outcomes over time to find a suitable tradeoff. In this way, prioritarianism points the way to an empirical approach to the ethics of risk regulations.

Responsibility plays a central role in responsibility-catering prioritarianism since the moral value of an outcome is dependent on considerations of this value. To give an example, if you and I are equally well-off, but I have brought about a risk that affects us both equally, then responsibility-catering prioritarianism implies that it is morally right that I carry the cost of protecting against the risk. This argument suggests that one can take the considerations of Cranor into account without turning to non-consequentialism. It also suggests that the theory can take essential ethical values into account better than utilitarianism. However, the value of responsibility raises several complicated issues that need more discussion than has yet been afforded them. It is time to turn to these issues now.

One initial attempt at constructing criteria for when responsibility should be taken into account for risk-regulations can be constructed from Carl Cranor’s list. First, a necessary condition of responsibility would be that an agent voluntarily takes a risk. Options that the agent are forced to take or risks that he or she cannot avoid are not the kind that infers responsibility. This condition is in line with Dworkin’s distinction between brute luck and option luck—it is only if the agent can exercise choice that the notion of option luck applies. However, it is important not to mistake the distinction between option luck and brute luck with a hard dichotomy because there can certainly be degrees of control. For example, having to take risky employment to survive can hardly be seen as entirely voluntary or consistent with a high degree of control.

It is, in this context, relevant to note that responsibility-catering prioritarianism can take responsibility into account on several levels. Compare the two cases of loss in well-being caused by on the one hand discrimination and the other hand the same loss of welfare caused by the impacts of a hurricane. In the first case, there is a perpetrator that should be held accountable, and it seems intuitively reasonable that the cost of rectifying discrimination should fall on the perpetrator of discrimination. In the second case, there is no perpetrator, and the question of responsibility is much more complicated. For example, if people have chosen to build houses in flood-prone areas despite warnings from authorities or if the local government has neglected the maintenance of flood protection systems or allowed people to build in dangerous zones. This example suggests that a more nuanced notion of responsibility can be relevant for thinking about risks regarding natural hazards.

A further issue is that the transparency and knowledge of risks and consequences will matter. For a choice to be informed, the agent must have access to information such that he or she can gain relevant knowledge concerning the situation in which the decision will be taken. This underlines the importance of risk information efforts as a part of risk management. A choice under the circumstance of unknown possibilities is not a deliberate gamble in Dworkin’s sense, and the outcomes of unknown options will consequently amount to brute luck. One should not be held responsible for not choosing something one did not know existed nor for exercising options that have characteristics that were unknowable at the time of choosing.

Therefore, a sufficiently high degree of control and knowledge of risks and consequences are necessary for considering an individual to be responsible. If the degree of control or knowledge is low, then responsibility should not be taken into account. It is only when the choice is voluntary, and the degree of control and knowledge of the consequences is sufficient, that responsibility should enter the calculation. However, the question of what should count as sufficient degrees of control and knowledge remains, and the final choice of whether or not to take responsibility into account in a particular social decision will have to depend on value-judgments on these two elements.

The argument up until this point has shown that results in political philosophy can be used to handle ethical issues regarding risk and that responsibility-catering prioritarianism is a plausible candidate for being an appropriate ethical theory of risk, but risk management does not only raise theoretical issues. Practical feasibility is also of paramount importance. The next section presents a proposal on how prioritarianism could be operationalized in a social choice framework and suggest how responsibility could be incorporated in this framework.

## Operationalizing the Theory

How could the theory of responsibility-catering prioritarianism be put to practical use to manage societal risks? Much work needs to be performed before methods and tools have been produced that can compete with established decision-support approaches such as cost–benefit analysis, but the argument that will be presented here aims to show that such methods seem feasible. For this purpose, the discussion here will mainly rely on the groundbreaking work of Matthew D. Adler (2008, 2009, 2012), who has developed a social choice framework, which, it will be suggested, can be used to operationalize responsibility-catering prioritarianism. Adler’s framework can be briefly summarized as follows.

Assume that a numerical measure of the well-being of an individual—*a utility measure—*can be defined. Let $u_{i} (x)$ be the utility for individual $i$ in outcome $x$. Outcome $x$ can then be described as a vector of individual utilities: $\left( {u_{1} (x), u_{2} (x), \ldots ,u_{N} (x)} \right)$. Let $W(x)$ be a function from a utility vector to a real number: $W(x) = W\left( {u_{1} (x), u_{2} (x), \ldots ,u_{N} (x)} \right).$ A ranking of two different outcomes $x$ and $y$ can then be constructed by defining outcome $x$ to be at least as good as outcome $y$ if and only if $W(x) \ge W(y)$. If different choice alternatives yield different outcomes, the alternative with the highest-ranking outcome is then chosen. The social welfare function (SWF) is simply the mathematical rule for ranking different outcomes, $W(x)$.

Given this social choice framework, different SWFs can be defined. A utilitarian SWF that says that the outcome with the greatest sum of individual utilities will be ranked highest will be defined as $W(x) = \mathop \sum \nolimits_{i = 1}^{N} u_{i} (x)$. A prioritarian SWF will be of the form $W(x) = \mathop \sum \nolimits_{i = 1}^{N} g(u_{i} (x))$, where $g( \cdot )$ is a strictly increasing and strictly concave function. This means that benefits to worse off individuals will be given greater weight in the calculation. For example, a prioritarian SWF could be $g\left( {u_{i} (x)} \right) = \sqrt {u_{i} (x)}$ (a strictly increasing and concave function). Assume that there are only two individuals with $u_{1} = 3$ and $u_{2} = 1$, and one has to choose between Policy A that increases the utility of individual 1 with one unit and Policy B that increases the utility of individual 2 by one unit. A prioritarian SWF would then rank the Policy A to be better than the Policy B (because $\sqrt 4 + \sqrt 1$ < $\sqrt 3 + \sqrt 2$), while a standard utilitarian SWF would be indifferent between Policy A and B.

If the prioritarian and utilitarian SWFs described above are going to be meaningful, one needs a utility measure $u( \cdot )$ that is *interpersonally comparable*. This means that it should be possible to say that the well-being of individual $i$ is greater than the well-being of individual $j$. One also needs to specify the function $g( \cdot )$ that provides the prioritarian weighing of the SWF. Exactly how the utility measure $u( \cdot )$ and prioritarian weighing function $g( \cdot )$ should best be defined is outside the scope of this article, but note that the feasibility of interpersonally comparable utilities is a common assumption in the literature on moral philosophy and social welfare functions. Adler’s (2012) approach to creating a utility measure builds on John Harsanyi’s (1986) idea of *extended preferences*. For the prioritarian weighing function, Adler argues for a set of continuous and prioritarian SWFs commonly used in welfare economics, which are called Atkinsonian SWFs. A simple Atkinsonian SWF is $W(x) = \frac{1}{1 - \gamma }\sum\nolimits_{i = 1}^{N} {u_{i}^{1 - \gamma } }$, where $\gamma$ is an “inequality-aversion” parameter with $\gamma > 0$.

The choice of $\gamma$ determines the amount of equality-sensitivity of *W* (it goes towards utilitarianism as it approaches zero, while it becomes an absolute priority for the worst-off individual as it approaches infinity). How inequality-averse the social policy should be (what value should $\gamma$ have) is an inherently normative question.

The final, and central element is a way to incorporate responsibility into this framework. Several different approaches to modeling considerations of responsibility in social choice frameworks have been developed recently (for an overview see Fleurbaey 2008, chapter 8, see also Adler 2012, pp. 579–584). It is beyond the scope of this paper to compare and assess which approach to model responsibility is the best. Instead, the aim here is only to argue that it is indeed possible to operationalize responsibility in a prioritarian social choice framework.

The proposal described here is based on defining a “hypothetical utility” that is used in the calculation of the social welfare function (the proposal is similar to, but distinct from Adler’s proposal). More specifically, the proposal here is that responsibility can be modeled by introducing a hypothetical utility measure called *responsibility*-*adjusted utility*, defined as follows:

responsibility-adjusted utility, $v_{i} \left( x \right)$ = the utility individual $i$ could normally be expected to achieve, given circumstances affecting $i$ that are outside of $i$:s control.

The responsibility-catering prioritarian social welfare function then becomes $W(x) = \sum\nolimits_{i = 1}^{N} {g\left( {v_{i} (x)} \right)}$. The social ranking of the outcomes $x$ and $y$ is done as before: $x$ is at least as good as $y$ if and only if $W(x) \ge W(y)$. The interpretation of the relationship between the expected utility, $u_{i} (x)$, and the responsibility-adjusted utility, $v_{i} (x)$, is the following: If $u_{i} (x) > v_{i} (x)$, then individual $i$ is seen as responsible for achieving a higher utility than could normally be expected. The implication is that individual $i$ is treated as if she or he had a *lower* utility than in reality, which will be an *advantage* for $i$ in the evaluation of polices that assign priority to the worst off. If, on the other hand, $u_{i} (x) < v_{i} (x)$, then individual $i$ is considered responsible for achieving a lower utility than could normally be expected. Individual $i$ is then treated as if she or he had a *higher* utility than in reality, which will be a *disadvantage* for $i$ in the evaluation of polices that assign priority to the worst off.

The interpretation of $v_{i} (x)$ above states that it should be set relative to circumstances outside the control of the individual. This interpretation means that different individuals may have various responsibility-adjusted utilities, given different circumstances. What is called circumstances correspond to “brute luck” in Dworkin’s terminology (see “Introducing Responsibility-Catering Prioritarianism” above). Precisely what should count as circumstances and how they should influence $v_{i} (x)$ depends on the particular application (see Fleurbaey (2008) for a discussion on circumstances and non-circumstances concerning responsibility).

The proposed approach is perhaps easier to understand by examples comparing the results of the social welfare function for normal utility-numbers and responsibility-adjusted utilities. This approach will, therefore, be illustrated by hypothetical examples of health policy decisions that are more or less caused by personal risk-taking. A commonly used example when discussing responsibility will be used: tobacco smoking. It is, of course, controversial whether it is a mainly free choice to take up the habit of smoking, or if it is primarily due to a biological disposition or social and economic circumstances. Also, it should be stressed that this should not be taken as an argument that smokers ought to be treated in any particular way, but only as an example used as a basis for discussing how responsibility can be modeled by a social welfare function. This example should be relevant to the topic of this paper as it involves managing risks from the perspective of the policy-maker. In the following examples, it is assumed that the decision-maker has access to the expected utility of different alternatives, which by the way is not an unreasonable assumption in the case of exposure to statistically significant health risks in large populations, such as smoking.

To make the example as simple as possible, calculations will be made for only two individuals: Jill who is a smoker and Jack who is a non-smoker. The smoker, Jill, is expected on average to have a shorter life and hence a lower level of well-being than the non-smoker, Jack. (Jack and Jill could be seen as representing a statistically significant group of individuals, but the examples become more accessible if one just assume that there are two individuals.) The outcomes for the two policy alternatives and the status quo are given in utility numbers (*expected utility* ($u_{i}$)) that allow for interpersonal comparisons of well-being levels, well-being differences, and comparisons to a neutral level of zero well-being.

The first example is as follows: Assume that the expected utility for Jack and Jill is 90 and 70 respectively without a policy (the status quo). A choice can be made between the Status quo or either of two alternatives: Policy A, which is a general health care program that improves the well-being of both individuals with equal amounts (+ 4 units), while Policy B focuses on smoking-related diseases and therefore only promotes the well-being of the smoker (+ 8 units). This example is summarized in Table 1.

**Table 1:** Numbers represent expected utility ($u_{i}$)

	Jack (non-smoker)	Jill (smoker)
Status quo	90	70
Policy A (Jack + 4, Jill + 4)	94	74
Policy B (Jack + 0, Jill + 8)	90	78

Which alternative (Policy A, Policy B, or Status quo) should be chosen? It is easy to see that a prioritarian social welfare function $W(x) = \mathop \sum \nolimits_{i = 1}^{N} g\left( {u_{i} (x)} \right)$, where $g( \cdot )$ is a strictly increasing and strictly concave function, would evaluate Policy B as better than Policy A.

For the sake of the example, assume that people who smoke should be considered responsible for smoking and the effects of smoking and that this should influence the choice of health care options. For this purpose, a similar table containing responsibility-adjusted utilities ($v_{i}$) can be constructed. For instance, if the average expected utility for non-smokers is $u = 90$, it could be argued that this is what should normally be expected, $v = 90$. If Jack and Jill are seen as belonging to the same class of relevant circumstances, the responsibility-adjusted outcomes will look like in Table 2.

**Table 2:** Numbers represent responsibility–adjusted utility ($v_{i}$)

	Jack (non-smoker)	Jill (smoker)
Status quo	90	90
Policy A (Jack + 4, Jill + 4)	94	94
Policy B (Jack + 0, Jill + 8)	90	98

In Table 2, Policy A would be preferred to Policy B from the point of view that gives sufficient priority to the worst off. The interpretation of the responsibility-adjusted utilities is that the difference between Jack and Jill is due to responsibility (they have the same circumstances). Hence, the preferred social policy is the one that does not take into account the lower expected well-being of Jill because she is seen as entirely responsible for smoking.

What happens if differences in circumstances between Jack and Jill are caused by factors outside their control (i.e., they are lucky or unlucky)? For example, Jill may be born with a handicap that reduces her general level of well-being. If the average utility for non-smokers with that typical handicap is 80, then the responsibility-adjusted utilities would instead be as in Table 3.

**Table 3:** Numbers represent responsibility–adjusted utility ($v_{i}$)

	Jack (non-smoker)	Jill (smoker)
Status quo	90	80
Policy A (Jack + 4, Jill + 4)	94	84
Policy B (Jack + 0, Jill + 8)	90	88

In Table 3, Policy B is the preferred choice according to a prioritarian evaluation. The effect of assigning Jack to a circumstance class with higher responsibility-adjusted value (e.g., 100) will be similar. The examples show how responsibility changes the evaluation when a responsibility-adjusted utility is used in a prioritarian social welfare function. This section has aimed to show, then, how responsibility-catering prioritarianism could be operationalized, although much work remains to elaborate all the details.

## Concluding Discussion

In this article, it has been argued that responsibility-catering prioritarianism is well equipped to handle the basic requirements of an ethical theory of risk since it takes not only efficiency but also equity and responsibility into account. Factors that should influence when and how people should be considered responsible for social decisions on risk have been suggested. In particular, it has been proposed that voluntarily taken risks might create a responsibility that needs to be taken into consideration, but responsibility depends on the degrees of control as well as knowledge of the risks and consequences. Finally, it was shown how a decision-making framework based on social welfare functions could be used to model responsibility-catering prioritarianism by using a hypothetical utility measure called “responsibility-adjusted utility” in a prioritarian social welfare function framework.

This paper has indicated the first steps toward a theory of responsibility catering prioritarianism for risk management, but more work remains. The question of when and how responsibility ought to matter is a complex moral issue, and there might be classes of decisions that should not be sensitive to responsibility. For example, it could be argued that health care for smokers is precisely a policy area where responsibility should not matter because health care should be equally available to all.

One possible way of arriving at how responsibility should influence policy areas can be created from John Rawls’s discussion of the basic structure of society, in which the infrastructure of society is the background against which responsibility takes place and perhaps becomes possible. For example, it can be argued that no one deserves a specific form of energy system in society, and no responsibility factors come into play for the average person when it is investigated what energy system to implement. Similar arguments may be relevant for health care. Such considerations illustrate why responsibility-catering prioritarianism may imply much less harsh policies than one would expect at first glance. A risk-responsibility analysis of the Rawlsian theory of justice could, therefore, be an interesting topic for further research.

Questions also remain regarding the weighting of the priority of the less advantaged. Determining just the right amount of priority could gain from work of social scientists investigating so-called social preferences, as well as research of ethicists and political philosophers working on distributive issues. Hopefully, this article has motivated such projects and has shown that it is worthwhile to start off towards a responsibility-catering prioritarian ethical theory of risk.

## Footnotes

1 The nine aspects in the list are presented in a different order than in Cranor (2007) to make the points of the analysis clearer.

2 The leveling down problem can be explained as follows: If what one cares about is that each person has an equal amount of, say, money, then one is committed to the idea that it is better when each person has equally little (e.g., a dollar a day each) than when each person has more, if this means that the money is distributed unequally (say 100 and 102 dollars a day).
